# Supplementary material for: CRISPR-CasRx-mediated disruption of Aqp1/Adrb2/Rock1/Rock2 genes reduces intraocular pressure and retinal ganglion cell damage in mice
Source: Nat Commun. 2024 Jul 30;15:6395. doi: 10.1038/s41467-024-50050-4 (PMC11289368; doi:10.1038/s41467-024-50050-4)
Supplement: Supplementary file 1 — Supplementary Information [file 41467_2024_50050_MOESM1_ESM.pdf]

Supplementary Information

Gene therapy for glaucoma using a combination targeting multiple genes with CRISPR-CasRx

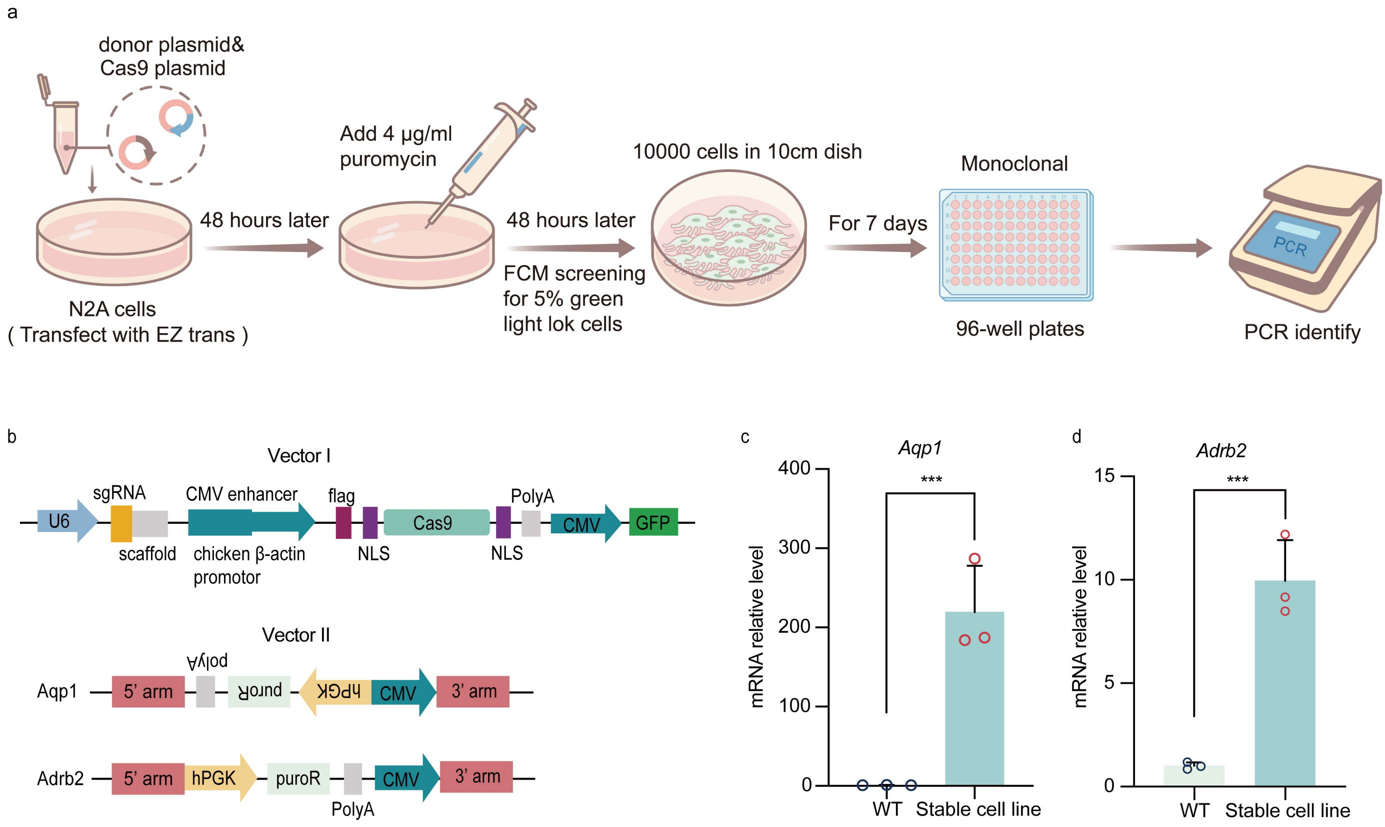

Fig. S1. Construct stable cell lines with high expression of *Aqp1*and *Adrb2* genes

a. Schematic diagram of constructing stable transfected N2a cell lines with high expression of target genes based on CRISPR-Cas9 knock in system. **b.** Plasmids for constructing stable cell lines, including sgRNA-Cas9 expression vector and vectors containing donor fragment. The donor fragment includes CMV promoter and puromycin resistance gene with 800bp homologous arms at both ends, which can be inserted precisely before the TSS sequence of mouse *Aqp1* gene or mouse *Adrb2* gene by homologous recombination. **c-d.** Compared with the wild type, the gene expression levels of *Aqp1* and *Adrb2*, in the established hybrid clone of stable transfected N2a cell lines were significantly increased, n=3. *Aqp1* mRNA levels were found to be up to 215-fold higher and *Adrb2* mRNA levels up to 9.85-fold higher compared to wild-type N2a cells. All values were expressed as mean ±SD, $*P < 0.05$, $**P < 0.01$, $***P < 0.001$, two-tailed, unpaired T-test. Source data are provided as a Source Data file.

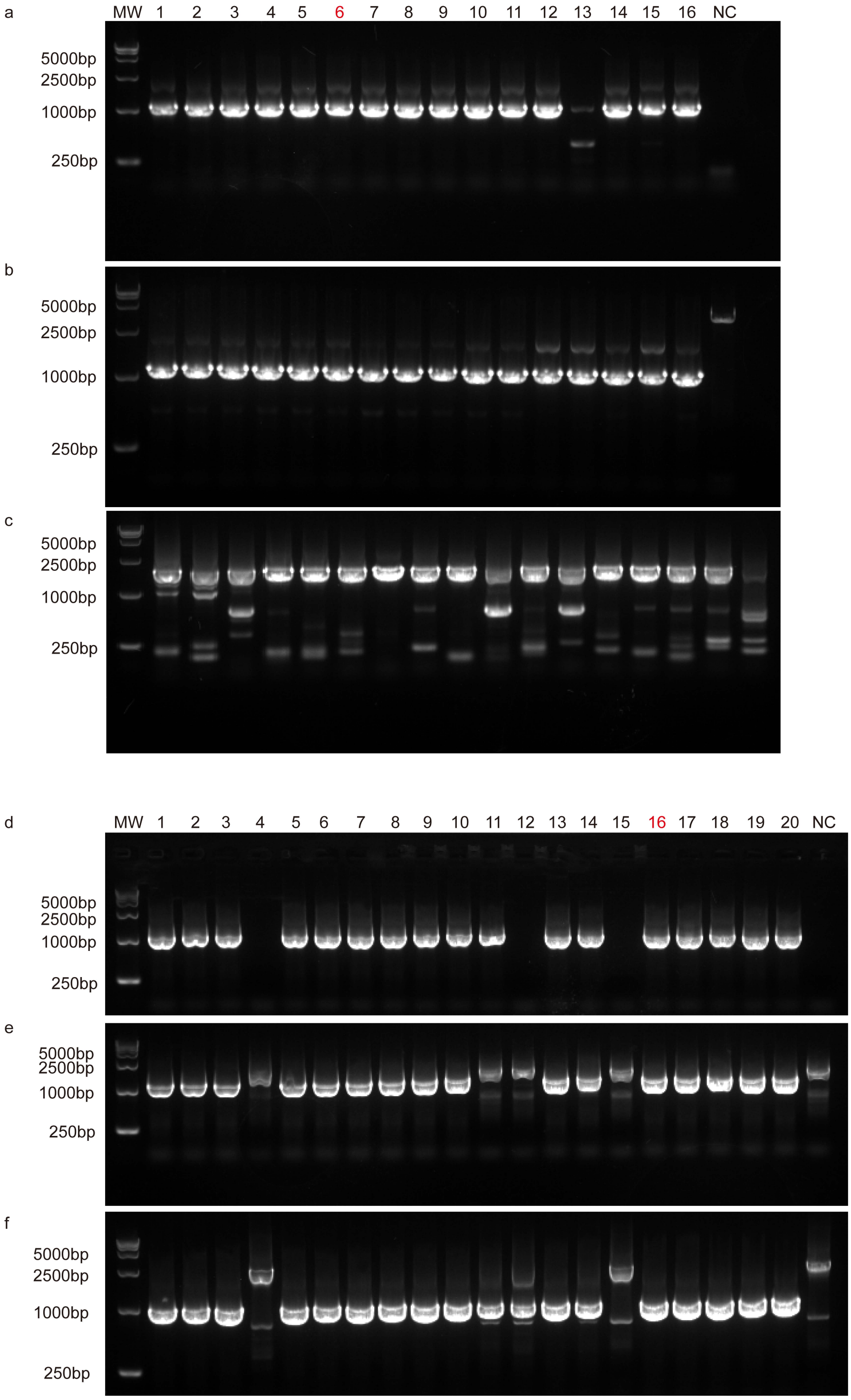

Fig. S2. N2a stable transfected cell monoclonal strains were identified by nested PCR.

a-c. Sixteen N2a stable cell monoclonal strains with CMV promoter inserted in front of *Aqp1* gene were identified by nested PCR and gel electrophoresis results were obtained. It shows the 5 'homologous arm side PCR identification results with a target band of 1068bp. PCR identification results of the knocked-in CMV promoter fragment in the b-diagram showed that the target band was 1739bp. **Fig. S2c** shows the PCR identification results of the 3 'homologous arm side with a target band of 1101bp. No. 6 was a high-expression Aqp1 monoclonal cell line with correct PCR identification and sequencing and finally sed for gRNA screening. **d-f.** Gel electrophoresis results of twenty N2a stable cell monoclonal strains with CMV promoter inserted in front of *Adrb2* gene were identified by nested PCR. Figure d shows the 5 'homologous arm side PCR identification results with a target band of 1399bp. PCR identification results of the knock in CMV promoter fragment were obtained in the **Fig. S2e**, and the target band was 1605bp. The **f**-graph was 3 'homologous arm side PCR identification with a target band of 1105bp. No. 16 was a monoclonal cell line with high expression of *Adrb2* that was correctly identified by PCR and sequencing and eventually screened by gRNA. MW: DL 15000 DNA marker; NC: negative control group.

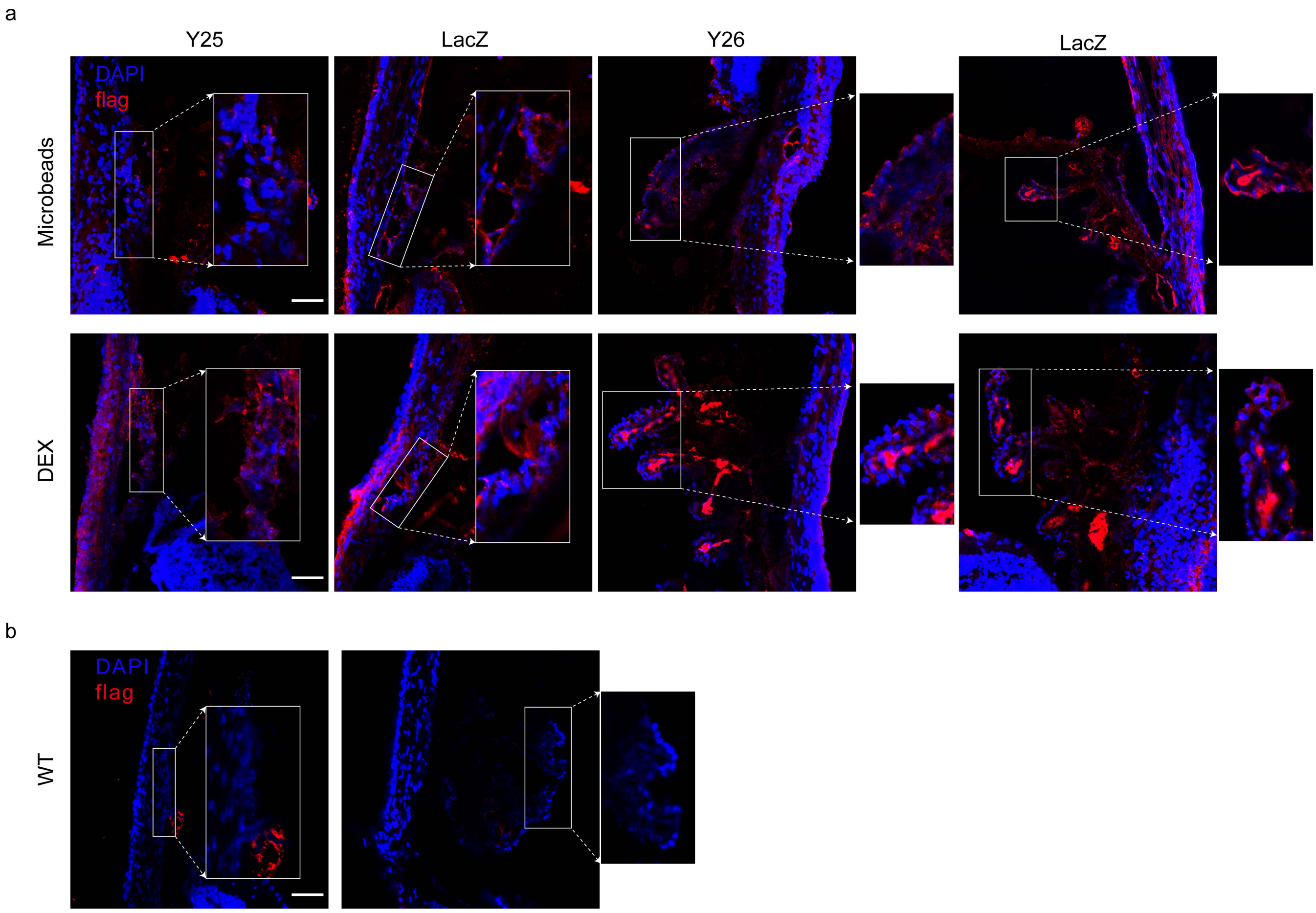

Fig. S3. The constructed shH10-Y25/Y26/LacZ virus can infect the trabecular meshwork and ciliary body of two types of glaucoma model mice.

a. The samples of magnetic beads-molded mice and DEX-induced mice were obtained 10 weeks after shH10 Y25/Y26/LacZ virus was injected into the glass cavity. The flag tag protein（red）of the virus was stained by immunofluorescence. A red fluorescence signal co-stained with DAPI was observed in trabecular reticulum in mice injected with shH10 Y25/LacZ virus. The red fluorescent signal co-stained with DAPI was observed in the ciliary body of mice injected with shH10 Y26/LacZ virus. *n*=3.Scale bars, 100μm. **b.** The samples of wild-type mice of the same age were stained by flag label protein immunofluorescence. No red fluorescence signal co-stained with DAPI was observed in trabecular reticulum and ciliary body. *n*=3. Scale bars, 100μm。

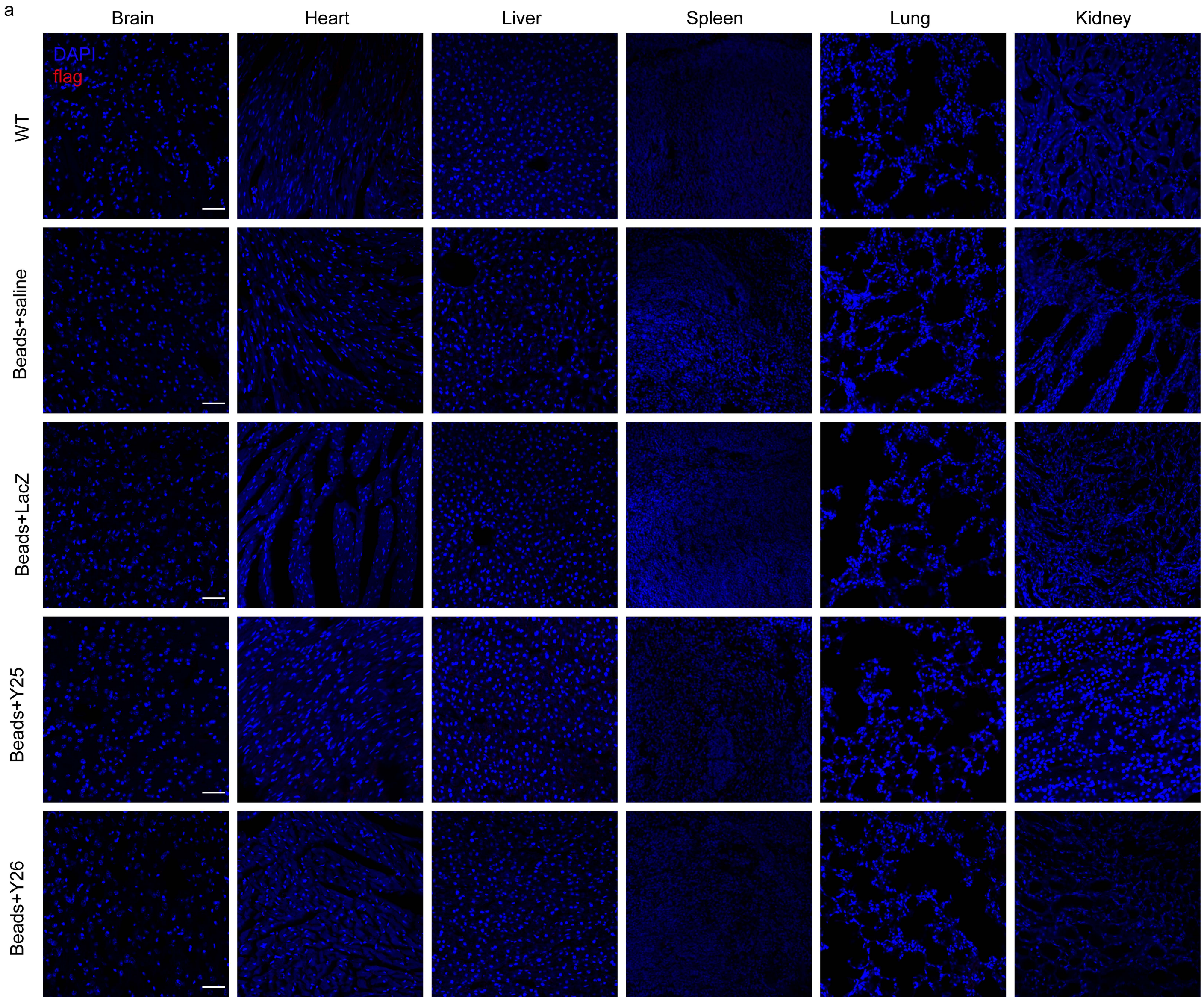

Fig. S4. Intravitreal injection of shH10 virus in magnetic beads-induced glaucoma mice is safe for multiple organs of the whole body.

a. Mice with magnetic beads-induced glaucoma were injected with shH10 LacZ/Y25/Y26 virus in the glass cavity, and the brain, heart, liver, spleen, lung and kidney were taken 10 weeks after modeling. The flag protein of the virus was stained by immunofluorescence. No red fluorescence signal co-stained with DAPI was observed. The results were consistent with those of mice with glaucoma induced by magnetic beads of saline injected into glass cavity and wild-type mice. *n*=3. Scale bars, 100μm.

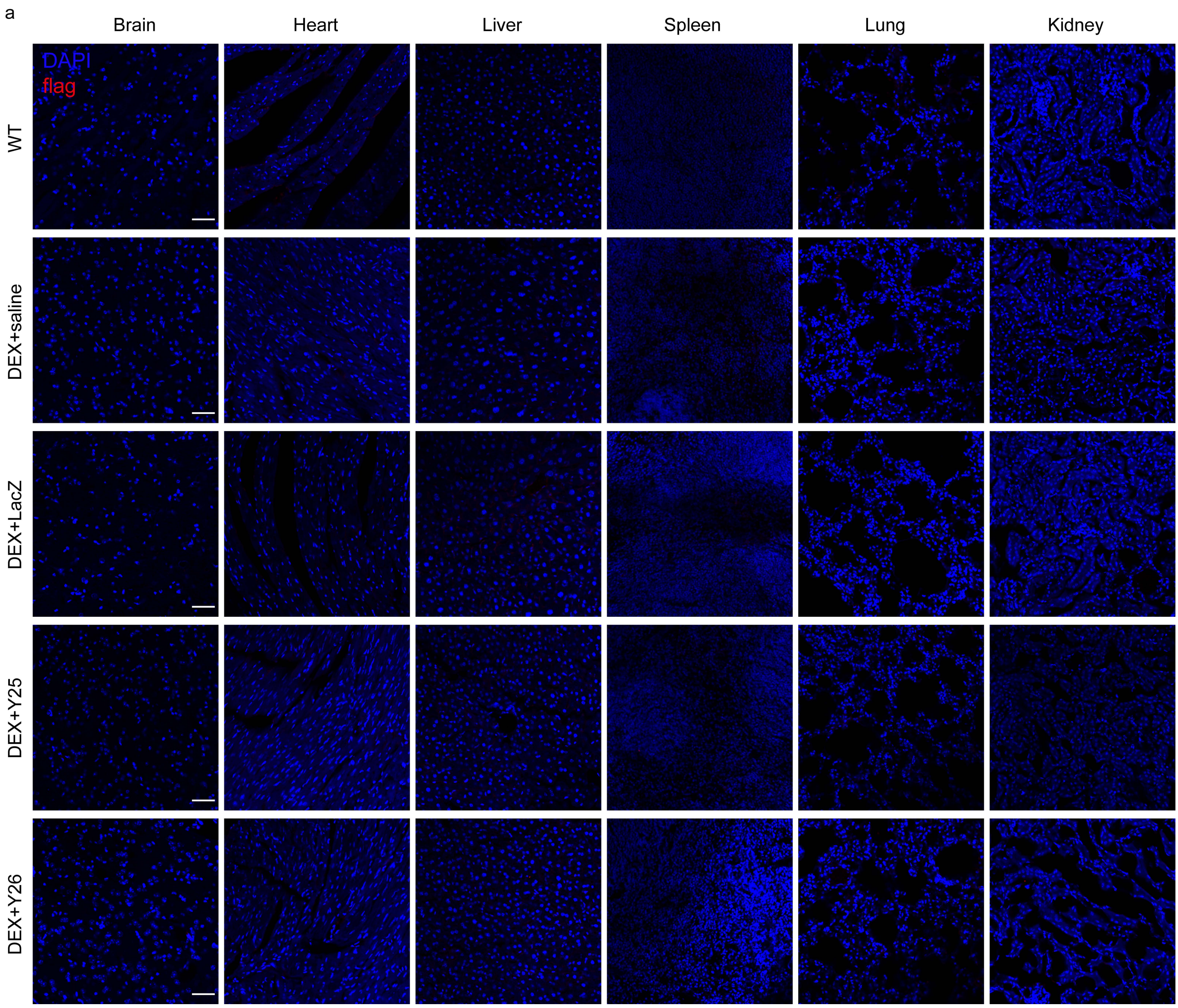

Fig. S5. Intravitreal injection of shH10 virus in DEX-induced glaucoma mice is safe for multiple organs of the whole body.

a. The DEX induced glaucoma mice were injected with shH10 LacZ/Y25/Y26 virus in the glass cavity. The brain, heart, liver, spleen, lung and kidney were taken 10 weeks after modeling, and the flag protein of the virus was stained by immunofluorescence. No red fluorescence signal co-stained with DAPI was observed， which was consistent with the results of wild-type mice and glaucoma mice induced by DEX injected with saline in glass cavity. *n*=3. Scale bars, 100μm.

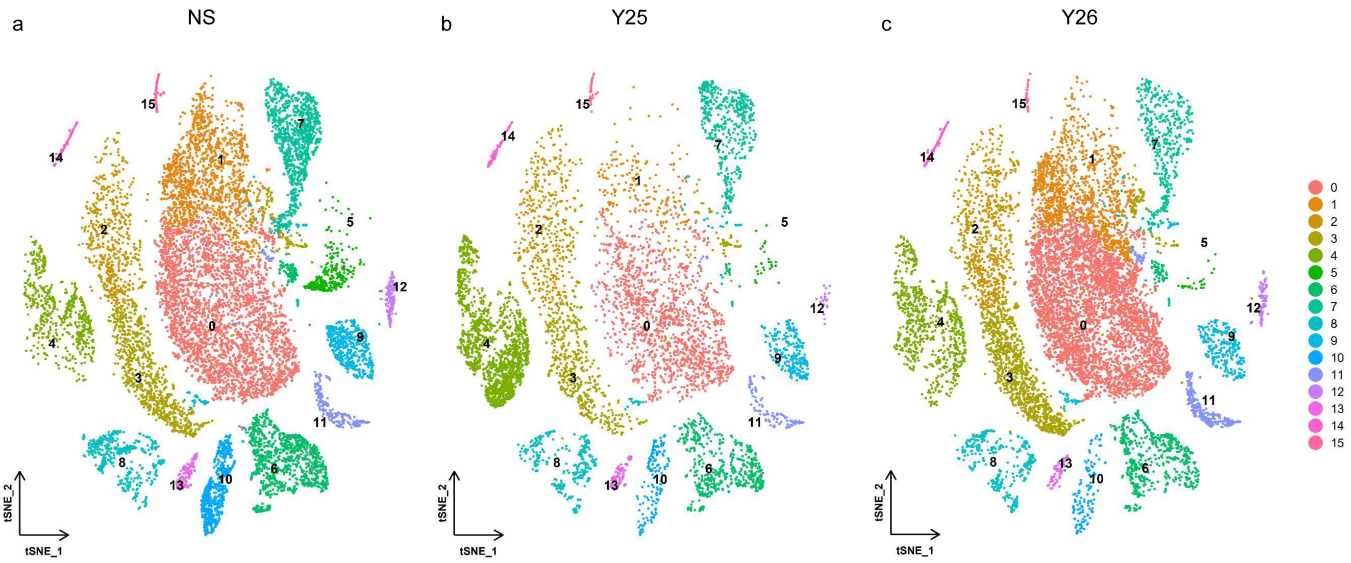

Fig. S6 The t-SNE plots of single-cell expression profiles from mouse CB and contiguous tissue.
a-c. The t-SNE plots showing the cell clusters from saline, Y25 and Y26.

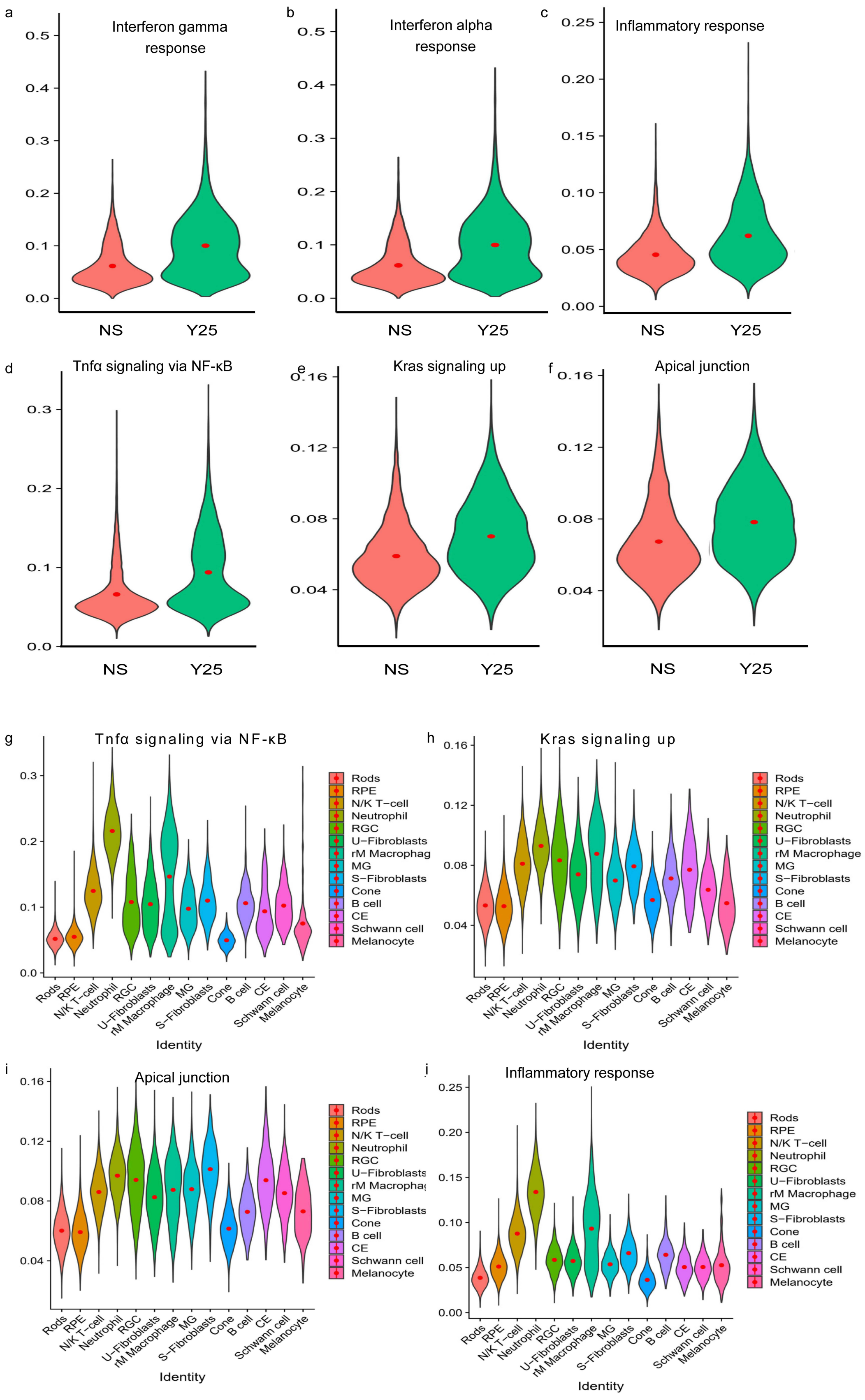

Fig. S7. The inhibition of *Rock1* and *Rock2* leads to a reduction in IOP by suppressing inflammatory responses.

a-f. The normalized enrichment scores (NES) of pathways which were up regulated by the inhibition of the *Rock1* and *Rock1* gene expression. **g-j.** The VlnPlot analysis of NES for each cell type revealed these signaling pathways may play a crucial role in the regulation of IOP.

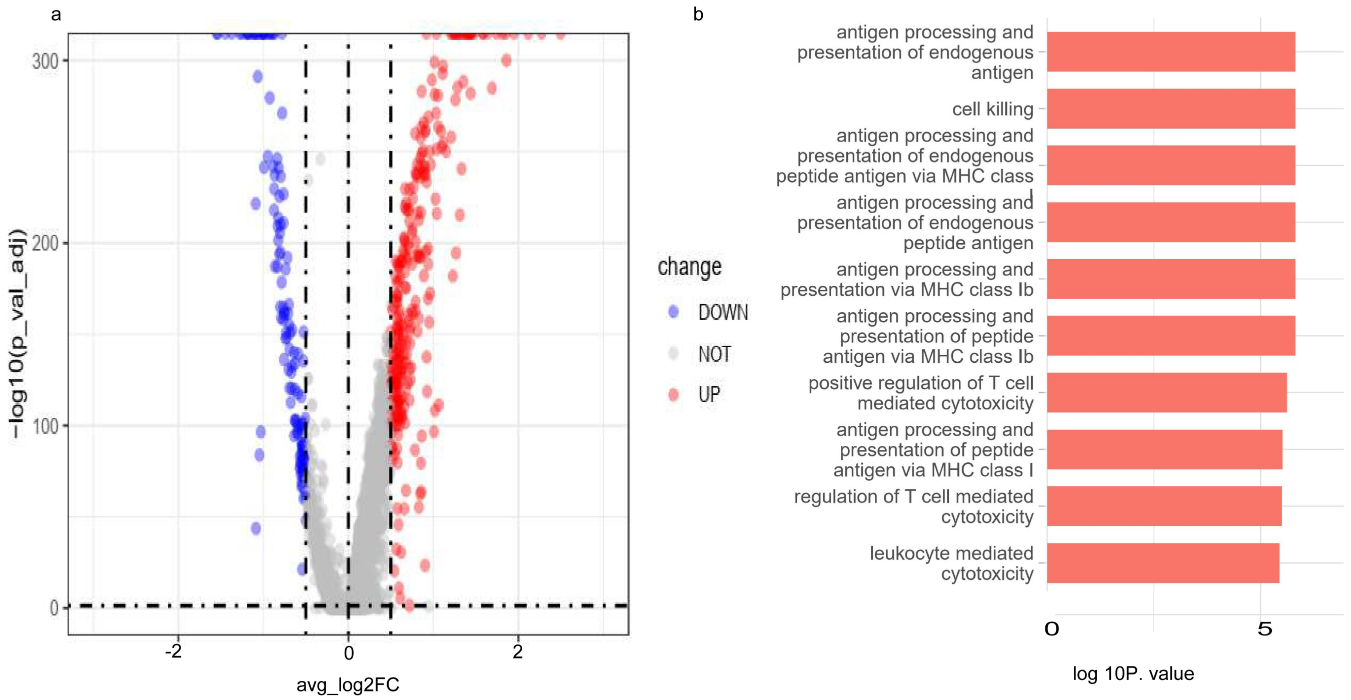

Fig. S8. The inhibition of *Rock1* and *Rock2* leads to a reduction in IOP by suppressing inflammatory responses.

a. The volcano map of differential genes after interference with *Rock1* and *Rock2* expression. **b.** The GSE analysis based on GO showed that the differential genes up-regulated in cells from Y25.

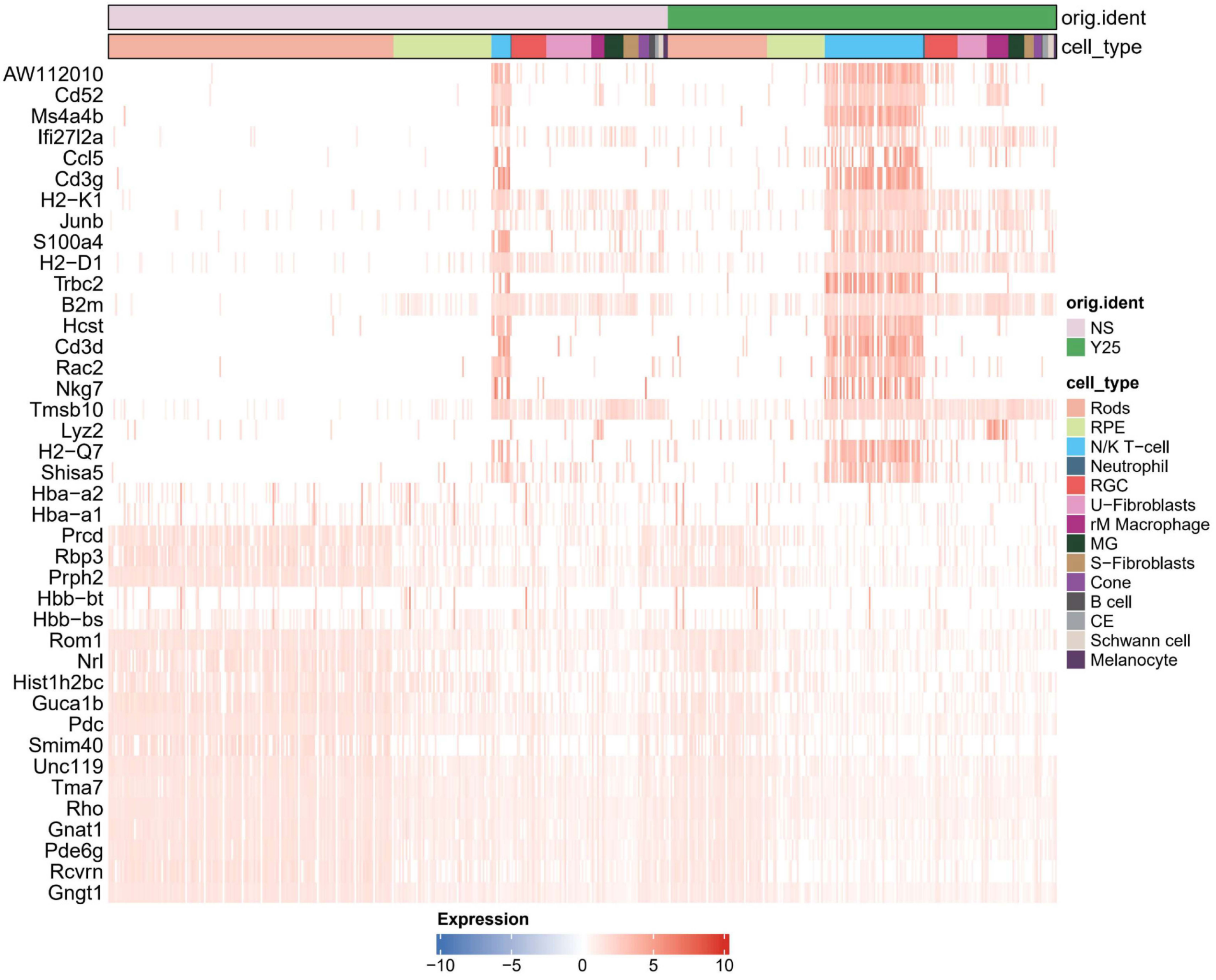

Fig. S9. The heatmap visualization of gene expression differences between shH10 Y25 and saline control displayed the top 20 differentially expressed genes (DEGs).

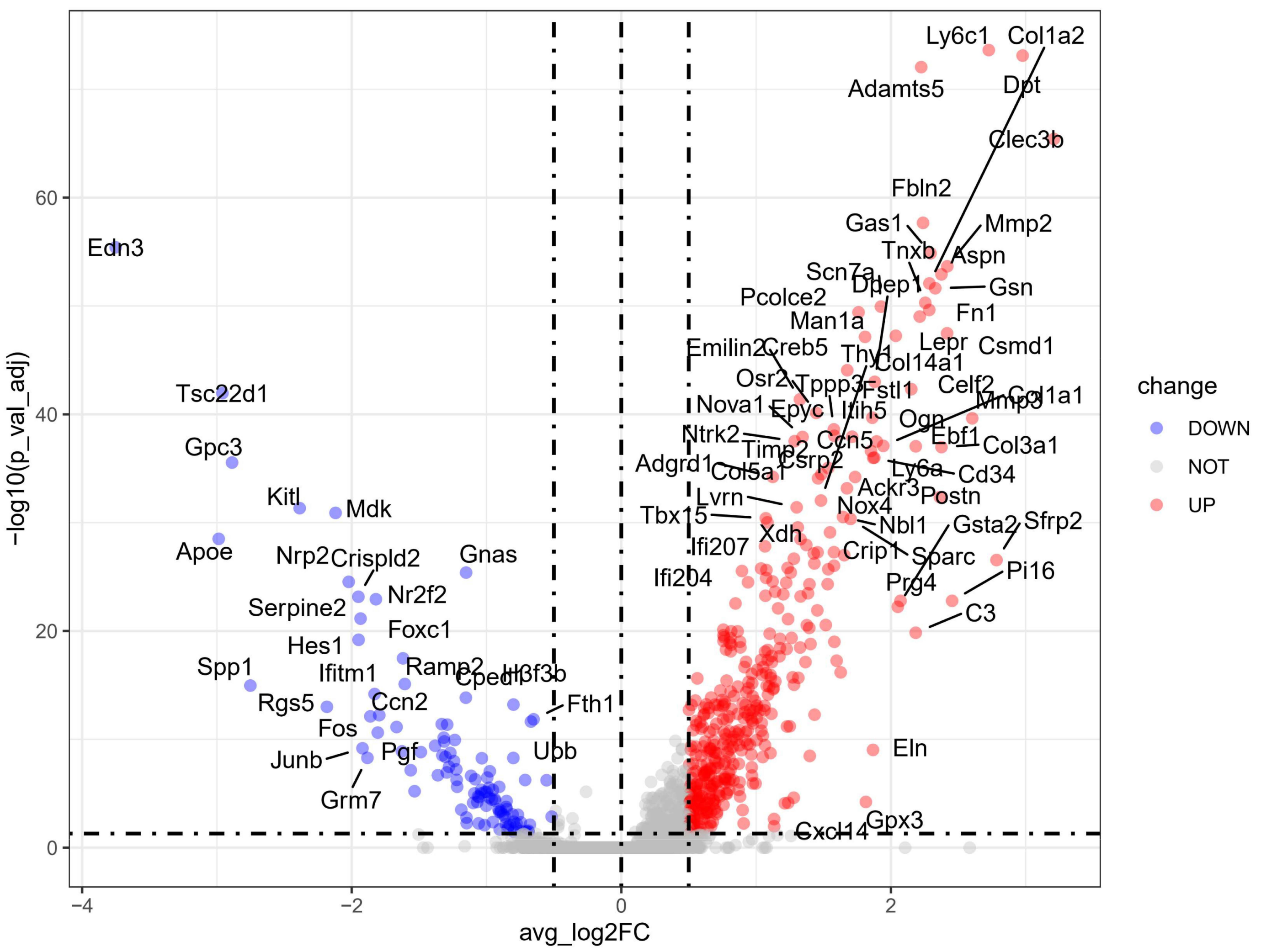

Fig. S10. The volcano map of differential gene between S-Fib and U-Fib from shH10.

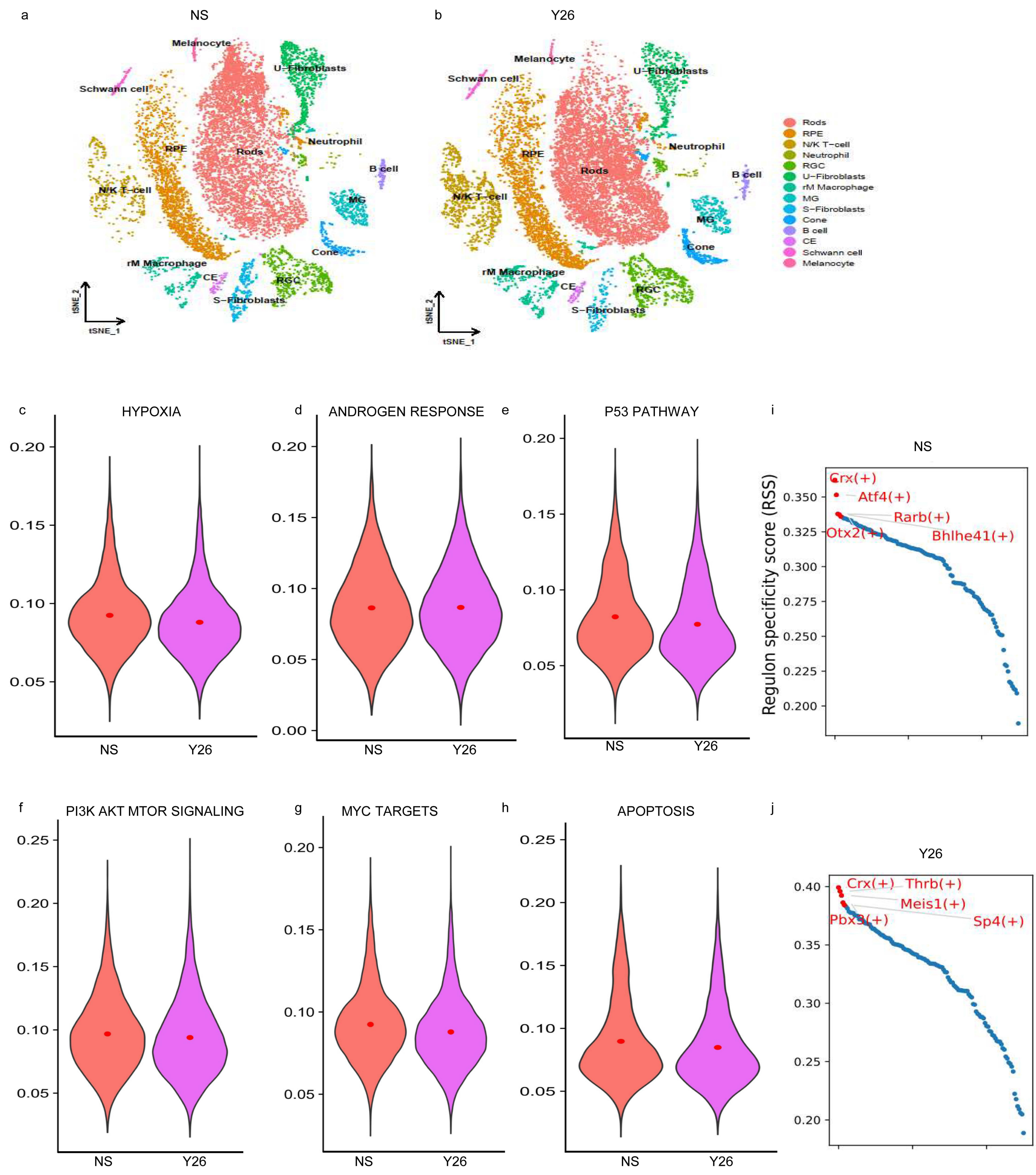

Fig. S11. The mechanism of downregulating the expression of Aqp1 and Adrb2 to attenuate IOP. **a.** The t-SNE plots showing cell type as determined by marker gene expression from shH10 Y26. **b.** The t-SNE plots showing cell type as determined by marker gene expression from saline control. **c-h.** The normalized enrichment scores (NES) of pathways which were up regulated by the inhibition of the *Aqp1* and *Adrb2* gene expression. **i-j.** SCENIC results of shH10 Y26 saline.

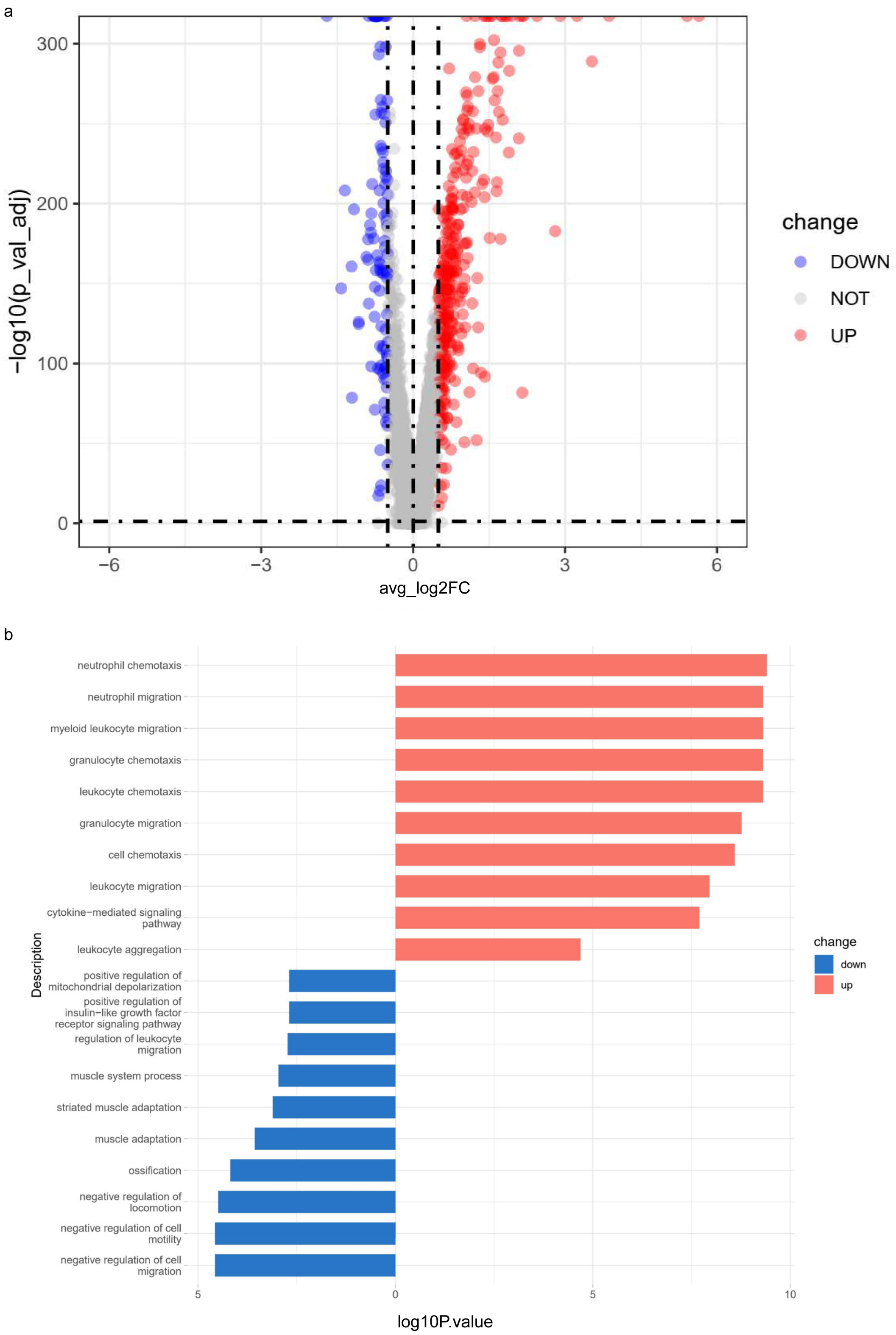

Fig. S12. The mechanism of downregulating the expression of *Aqp1* and *Adrb2* to decrease IOP.
a. The differential gene volcano map of inhibition of the *Aqp1* and *Adrb2* expression. **b.** The GSE analysis based on GO showed that the differential genes up-regulated in cells from Y26.

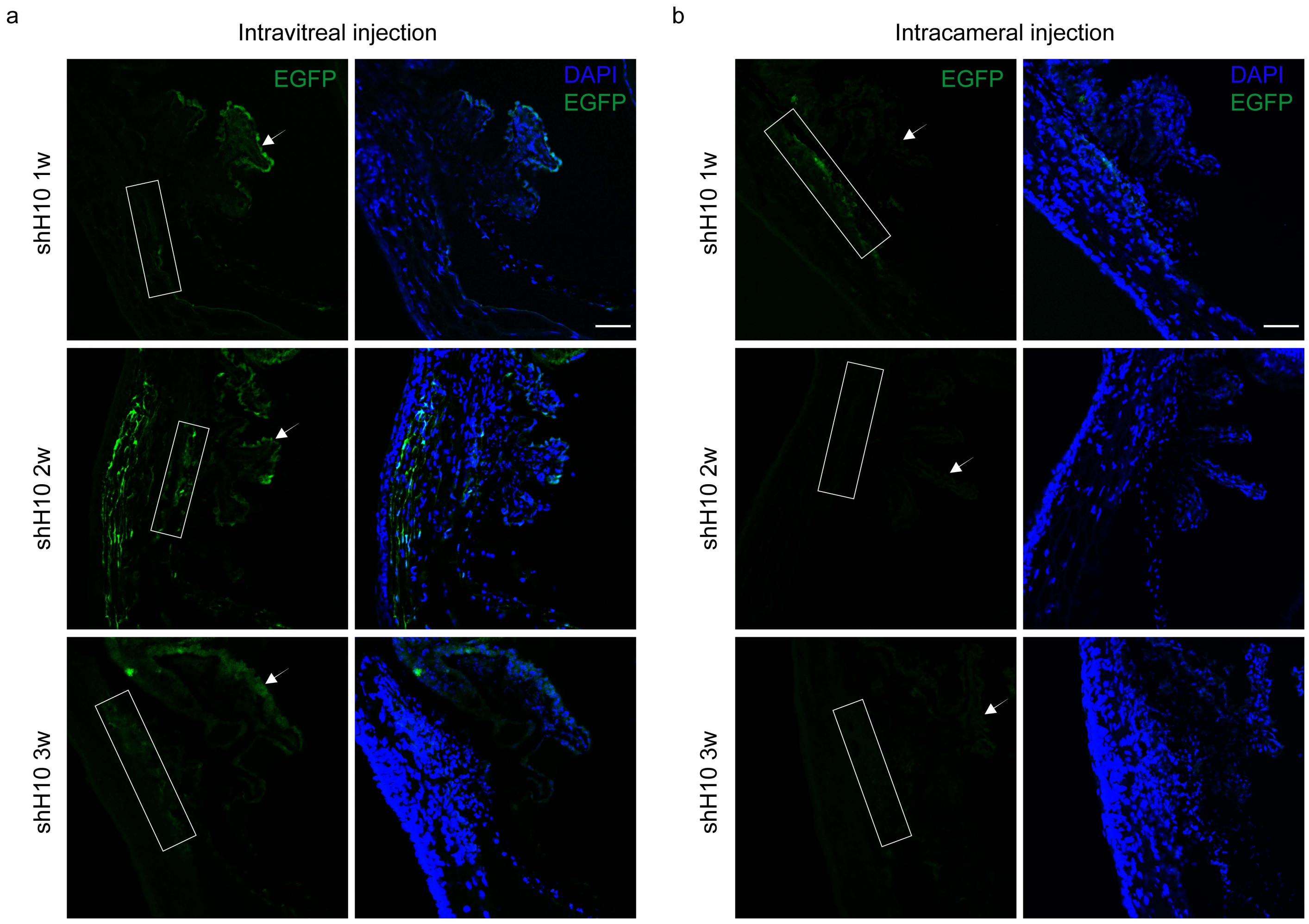

Fig. S13. Intraocular infection of shH10 virus by different injection methods.
The injected virus was shH10-EFS-EGFP with titer of 1E+13GC/ml and injection dose of 1.5μl. The eyeball was taken at one week, two weeks and three weeks after injection, respectively, and the expression of the virus in the CB and TM was observed. The arrow indicated the ciliary body and the box indicated the trabecular meshwork. **a.** Confocal image of ocular infection in mice after intravitreal injection of shH10 AAV virus. *n*=3.Scale bars,100μm. **b.** Confocal image of ocular infection in mice after intracameral injection of shH10 AAV virus. *n*=3.Scale bars,100μm.

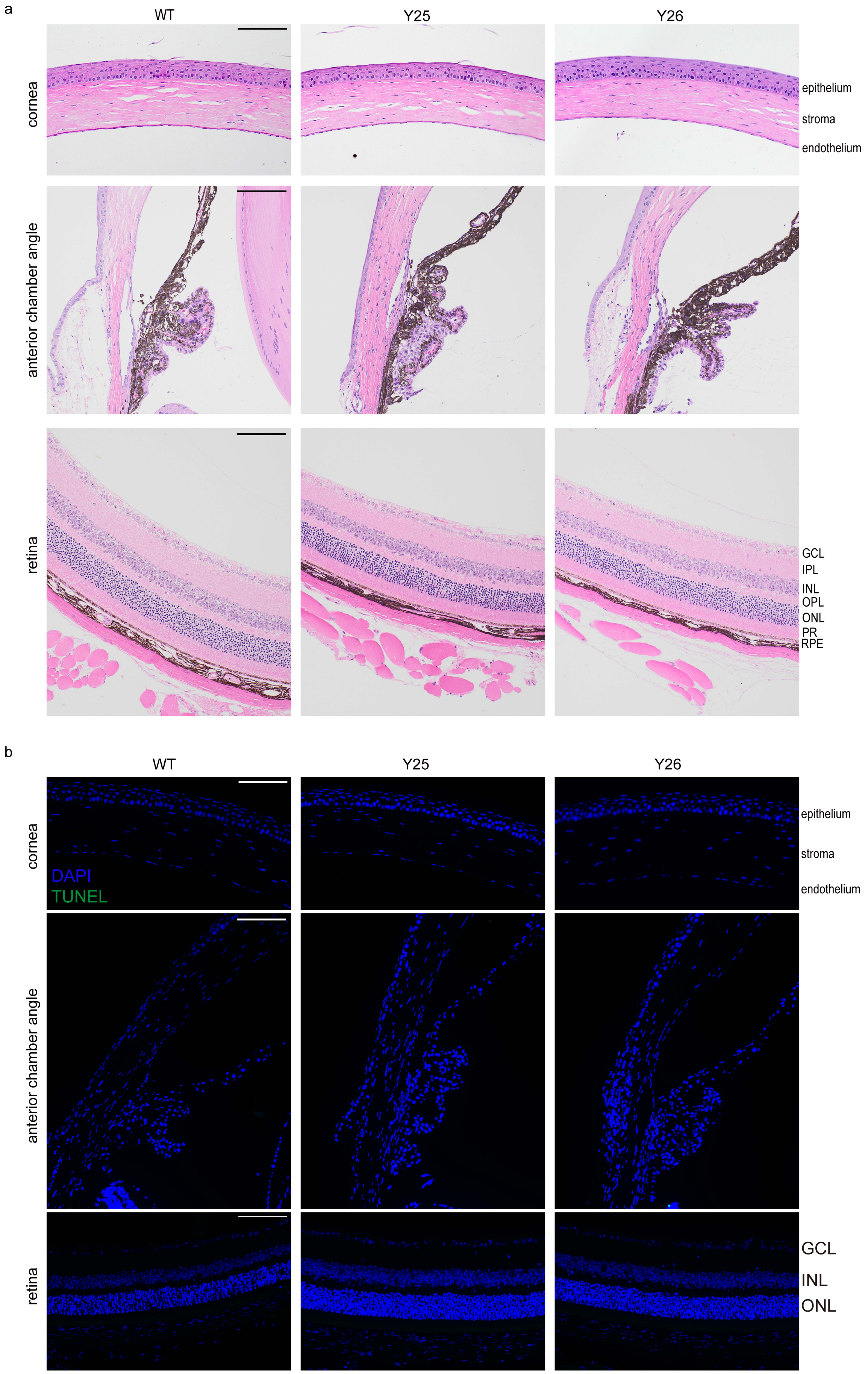

Fig. S14. Ocular safety of intravitreal injection of virus.

a. Hematoxylin-Eosin staining (H&E) of the cornea, anterior chamber angle and retina in mice intravitreal injected with shH10 Y25/Y26 virus for 10 weeks. WT: wild type mice. *n*=3.Scale bars,100μm. **b.** TUNEL test of cornea, anterior chamber angle and retina after being intravitreal injected with shH10 Y25/Y26 virus for ten weeks. WT: wild type mice. *n*=3.Scale bars,100μm.

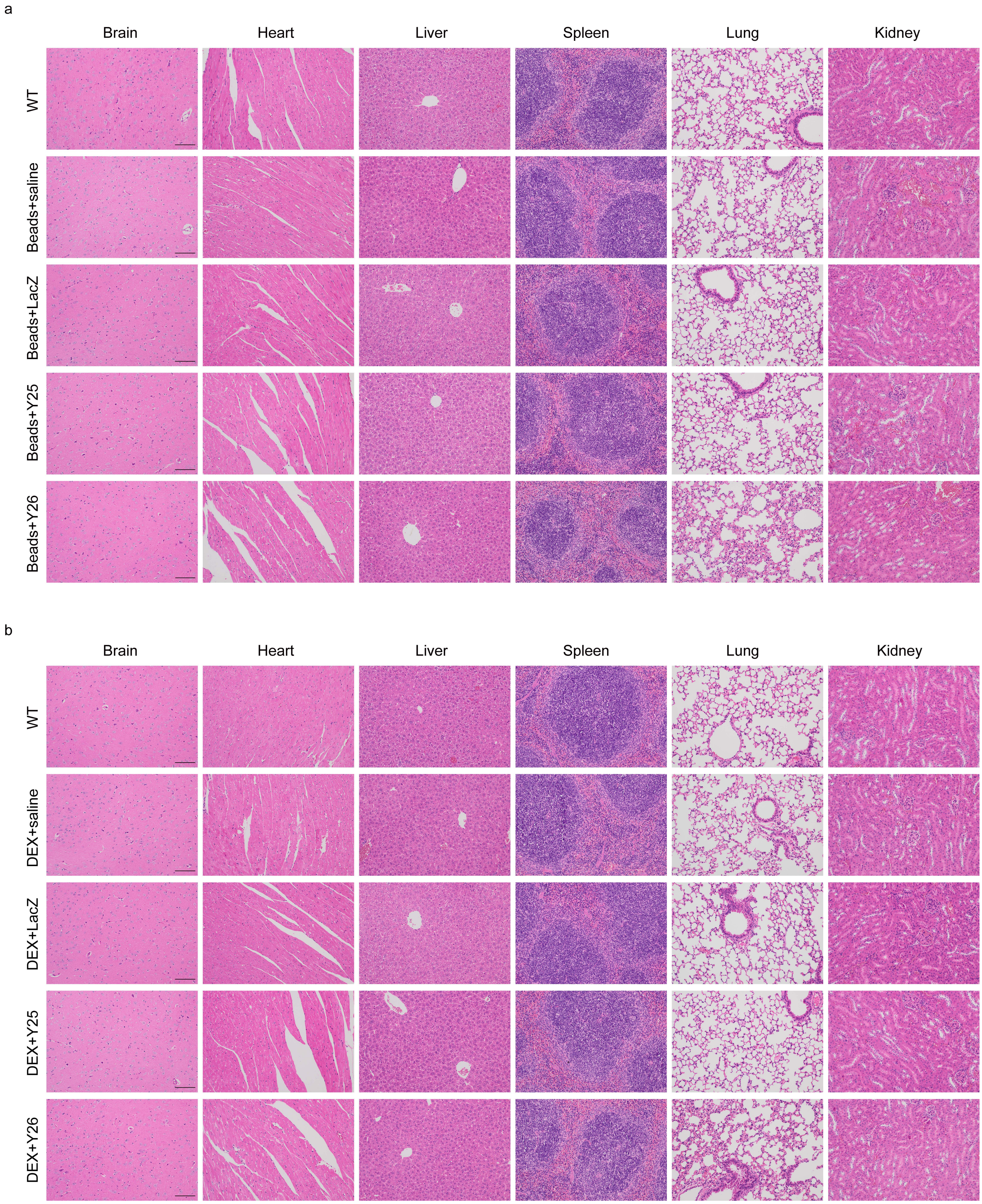

Fig. S15. Multi-organ safety of intravitreal injection of virus.

a. Hematoxylin-Eosin staining (H&E) of the different organs of the magnetic beads-induced model mice intravitreal injected with shH10 LacZ/Y25/Y26 virus. All samples were taken 10 weeks after modeling. WT: wild type mice. *n*=3.Scale bars,100μm. **b.** Hematoxylin-Eosin staining (H&E) of the different organs of the DEX-induced model mice intravitreal injected with shH10 LacZ/Y25/Y26 virus. All samples were taken 10 weeks after modeling. WT: wild type mice. *n*=3.Scale bars,100μm.

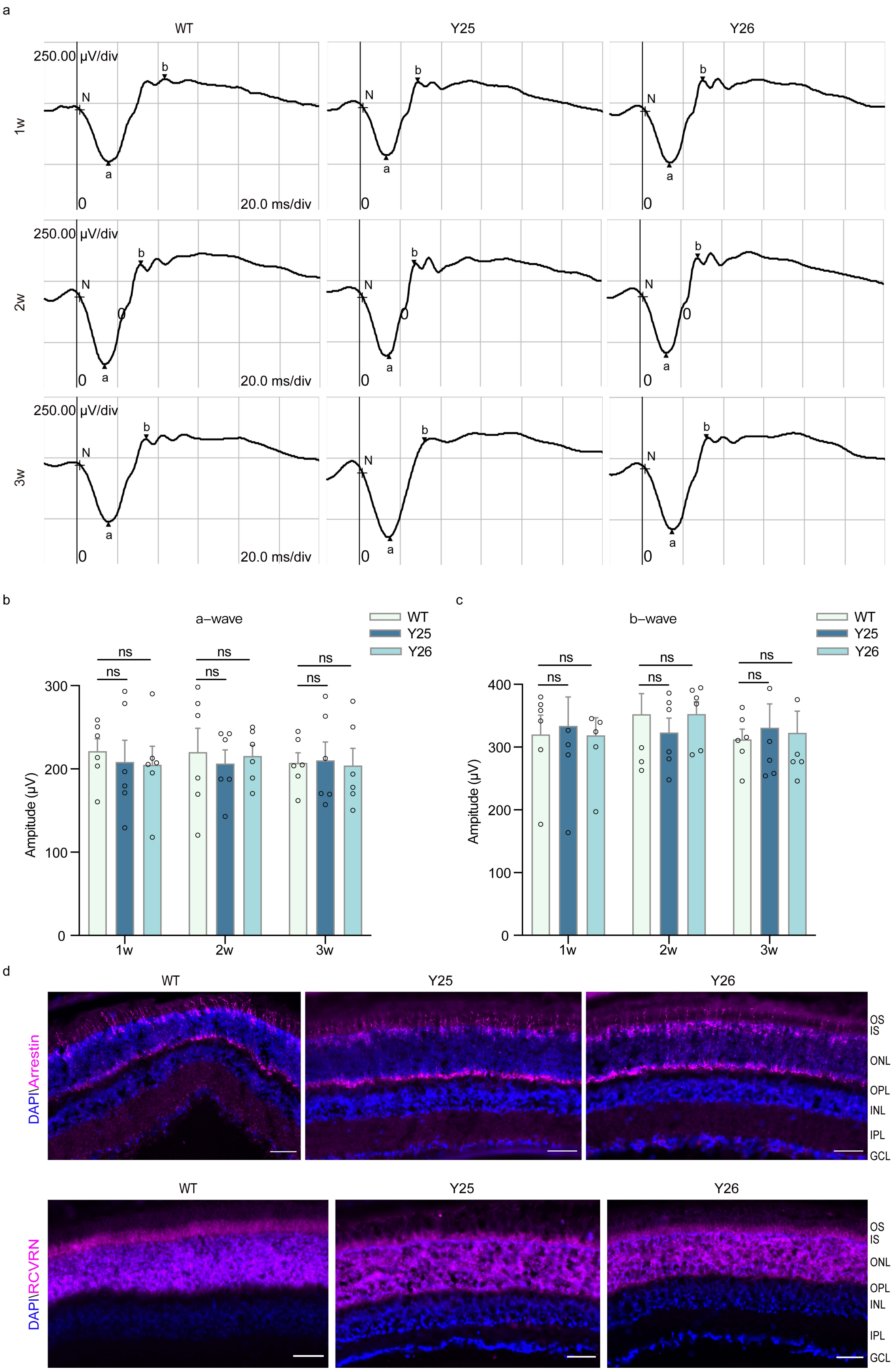

Fig. S16. Intravitreal injection of shH10 virus did not affect retinal photoreceptor function.
a. Full-field electroretinography (ERG) result of mice intravitreal injected with shH10 Y25/Y26 virus for 10 weeks. WT: wild type mice. **b-c.** Quantitative analysis of a-wave (**b**) and b-wave (**c**) amplitude. *n*=6. All values were expressed as mean ± SEM, two-tailed, unpaired T-test, ns, no significance. **d.** Immunofluorescence staining image of retinal photoreceptors of mice intravitreal injected with shH10 Y25/Y26 virus for 10 weeks. Arrestin labels cones. RCVRN labeled rod cells. WT: wild type mice. *n*=3.Scale bars,50μm. Source data are provided as a Source Data file.

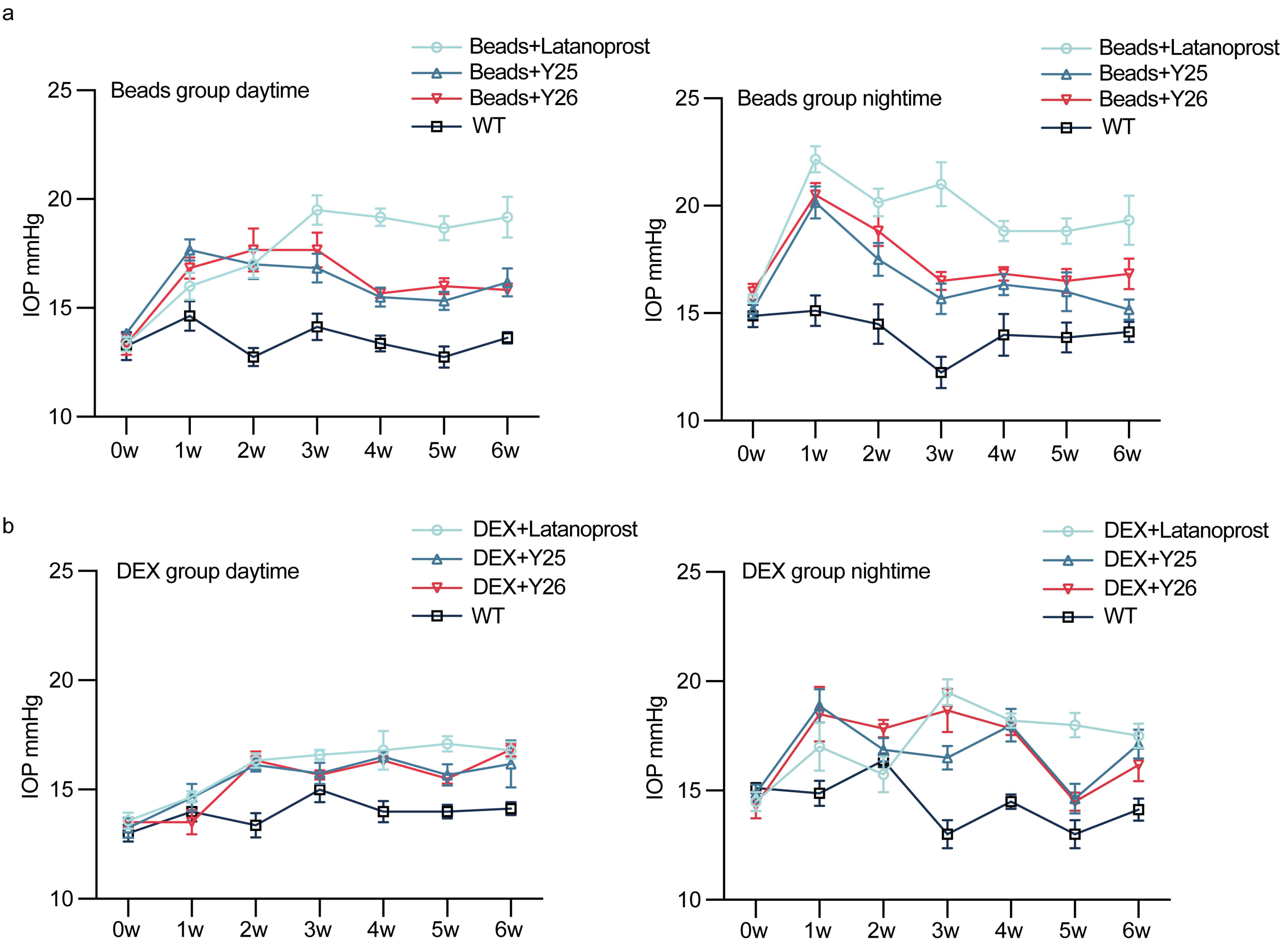

Fig. S17. The effect of AAV-delivered CasRx system on reducing IOP was compared with Latanoprost

a. Intraocular pressure (IOP) changed during day and night in each group after monocular magnetic beads modeling and injection of shH10 Y25, shH10 Y26 or using Latanoprost drops daily during six weeks. WT group *n*=8, Beads+Y25 group *n*=6, Beads+Y26 group *n*=6, Beads+Latanoprost group *n*=6, all values were expressed as mean±SEM, and differences between groups were tested by one-way ANOVA. **b.** IOP changed during day and night in each group after DEX modeling and injection of shH10 Y25, shH10 Y26 or using Latanoprost drops daily during six weeks. WT group *n*=8, DEX+Y25 group *n*=8, DEX+Y26 group *n*=6, DEX+Latanoprost group *n*=10, all values were expressed as mean ±SEM, and differences between groups were tested by one-way ANOVA. Source data are provided as a Source Data file.

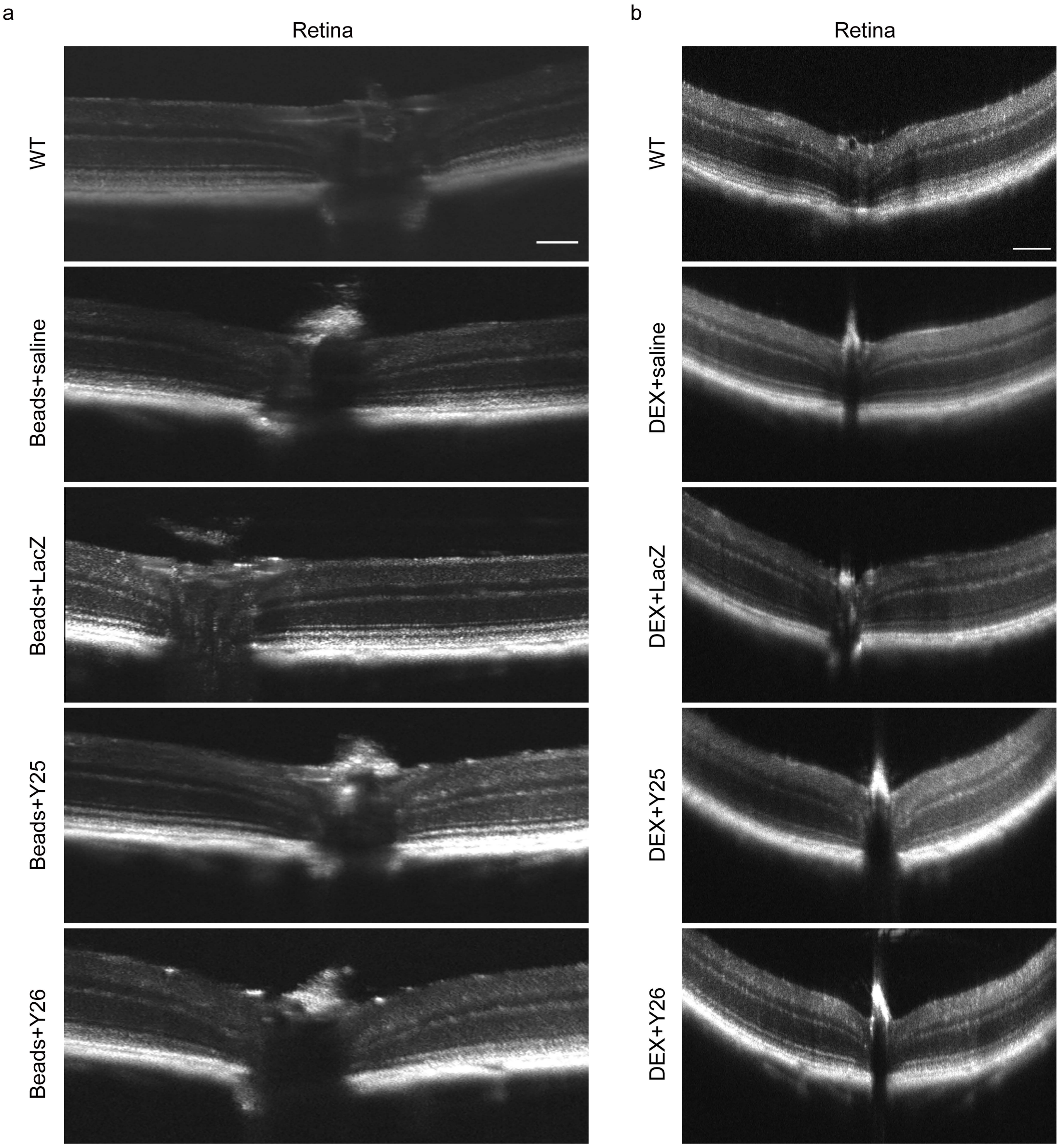

Fig. S18. OCT images of retina in two types of glaucoma models treated with shH10 Y25\Y26.
a. OCT image of retina in each group after monocular magnetic beads modeling and injection of shH10 virus for 10 weeks. *n*=4. Scale bars, 100μm. **b.** OCT image of retina in each group after DEX modeling and injection of shH10 virus. *n*=4. Scale bars, 100μm.

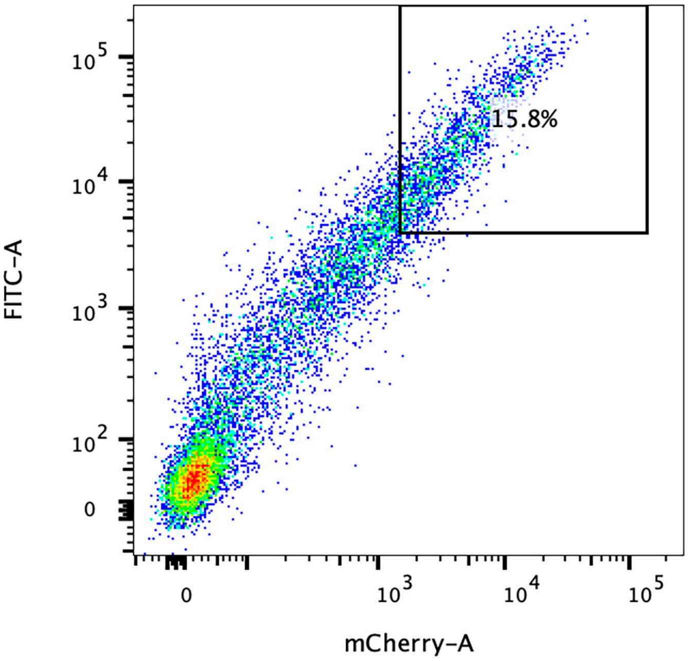

Fig. S19. Gating strategies for sorting N2a cells by fluorescence-activated cell sorting.

Table S1: List of gRNA sequences targeting different genes

ROCK1 gRNA-1 primer-F	ACAG agttgtcaatatttttgtttttcttaagg
ROCK1 gRNA-1 primer-R	CAGC ccttaagaaaaaacaaaaatattgacaact
ROCK1 gRNA-2 primer-F	ACAG gcatccaatgcaagaactacttctgcagta
ROCK1 gRNA-2 primer-R	CAGC tactgcagaagtagttcttgcattggatgc
ROCK1 gRNA-3 primer-F	ACAG gaataaaacccatggaatggattgcatcca
ROCK1 gRNA-3 primer-R	CAGC tggatgcaatccattccatgggttttattc
ROCK1 gRNA-4 primer-F	ACAG tacaagtaccaaaatctgctaacttcaaat
ROCK1 gRNA-4 primer-R	CAGC atttgaagttagcagattttggtacttgta
ROCK1 gRNA-5 primer-F	ACAG cttattcatcttcatacaagtaccaaaatc
ROCK1 gRNA-5 primer-R	CAGC gattttggtacttgtatgaagatgaataag
ROCK2 gRNA-1 primer-F	AAAC tggcaaaggccataatatctctttcttccc
ROCK2 gRNA-1 primer-R	CTTG gggaagaaagagatattatggcctttgcca
ROCK2 gRNA-2 primer-F	AAAC cttcagcaatggaacgagccagctgctcag
ROCK2 gRNA-2 primer-R	CTTG ctgagcagctggctcgttccattgctgaag
ROCK2 gRNA-3 primer-F	AAAC cacaggcaatgacaaccatccttctaatct
ROCK2 gRNA-3 primer-R	CTTG agattagaaggatggttgtcattgcctgtg
ROCK2 gRNA-4 primer-F	AAAC ttcgattcatgatctccgccaacttattca
ROCK2 gRNA-4 primer-R	CTTG tgaataagttggcggagatcatgaatcgaa
ROCK2 gRNA-5 primer-F	AAAC agcagtgtaaaacttggcccacttttcagg
ROCK2 gRNA-5 primer-R	CTTG cctgaaaagtgggccaagttttacactgct
ROCK2 gRNA-6 primer-F	AAAC gcccatagtaaccatcacctccttgtgatt
ROCK2 gRNA-6 primer-R	CTTG aatcacaaggaggtgatggttactatgggc
AQP1 gRNA-1 primer-F	AAAC gtggcgacgatggctcccacacactgggcg
AQP1 gRNA-1 primer-R	CTTG cgcccagtgtgtgggagccatcgtcgccac
AQP1 gRNA-2 primer-F	AAAC ccacagccctccagaagagcttcttcttga
AQP1 gRNA-2 primer-R	CTTG tcaagaagaagctcttctggagggctgtgg
AQP1 gRNA-3 primer-F	AAAC gggcagaaccaatgctgatgaagacgaaga
AQP1 gRNA-3 primer-R	CTTG tcttcgtcttcatcagcattggttctgccc
AQP1 gRNA-4 primer-F	AAAC tactgccccagacaaggctagaccatgtgg
AQP1 gRNA-4 primer-R	CTTG ccacatggtctagccttgtctggggcagta
AQP1 gRNA-5 primer-F	AAAC aggcacaaagcgacattggcagaatcccag
AQP1 gRNA-5 primer-R	CTTG ctgggattctgccaatgtcgctttgtgcct
AQP1 gRNA-6 primer-F	AAAC cttcatctccaccctggagttgatgtcgtc
AQP1 gRNA-6 primer-R	CTTG gacgacatcaactccagggtggagatgaag
ADRB2 gRNA-1 primer-F	AAAC tctctcctttagatatggatttccagcagc
ADRB2 gRNA-1 primer-R	CTTG gctgctggaaatccatatctaaaggagaga
ADRB2 gRNA-2 primer-F	AAAC tcatccagacagacagacagacagactcag
ADRB2 gRNA-2 primer-R	CTTG ctgagtctgtctgtctgtctgtctggatga
ADRB2 gRNA-3 primer-F	AAAC catgactagatcagcacacgccaaggagat
ADRB2 gRNA-3 primer-R	CTTG atctccttggcgtgtgctgatctagtcatg
ADRB2 gRNA-4 primer-F	AAAC cccatgactagatcagcacacgccaaggag
ADRB2 gRNA-4 primer-R	CTTG ctccttggcgtgtgctgatctagtcatggg

Table S2: List of primers for PCR identification and sequencing of inserted fragments

	**ADRB2 round 1**	
**5' arm**	ymy-89-1 F	ctcccatgcacagaacataa
	ymy-87-1 R	gggagagaggtcggtgattc
**insertfragment**	ymy-86-1 F	taagtcgggaaggttccttg
	ymy-104-1 R	tcaacggggcggggttatt
**3' arm**	ymy-103-1 F	tggcagtacaccaatgggc
	ymy-90-1 R	gcgtggaatcttccttcaga
	**ADRB2 round 2**	
**5' arm**	ymy-89-2 F	ctgaaaatggttgcacttgca
	ymy-87-2 R	ctccggaggcttgcagaat
**insertfragment**	ymy-86-2 F	aaacggaagccgcacgtctca
	ymy-104-2 R	tcccgttgattttggtgcca
**3' arm**	ymy-103-2 F	gggatttccaagtctccacc
	ymy-90-2 R	ttttggccacctggaagacc
	**AQP1 round 1**	
**5' arm**	ymy-85-1 F	ctcctctgctccgtttgtct
	ymy-100-1 R	agccatctgttgtttgcccc
**insertfragment**	ymy-101-1 F	cccaccccccagaatagaa
	ymy-104-1 R	tcaacggggcggggttatt
**3' arm**	ymy-103-1 F	tggcagtacaccaatgggc
	ymy-88-1 R	ggtagctggagaaaatgtactcg
	**AQP1 round 2**	
**5' arm**	ymy-85-2 F	cgtgctgttttttcctgtga
	ymy-100-2 R	ttgaccctggaaggtgccactc
**insertfragment**	ymy-101-2 F	ctcagacaatgcgatgcaatttcc
	ymy-104-2 R	tcccgttgattttggtgcca
**3' arm**	ymy-103-2 F	gggatttccaagtctccacc
	ymy-88-2 R	gactgcaaagggggaaggtg

Table S3：**List of primers used for qPCR**

AQP1-1 F QP-Mus	AGGCTTCAATTACCCACTGGA
AQP1-1 R QP-Mus	CTTTGGGCCAGAGTAGCGAT
ADRB2-1 F QP-Mus	ATGTCGGTTATCGTCCTGGC
ADRB2-1 R QP-Mus	GGTTTGTAGTCGCTCGAACTTG
ROCK1-1 F QP-Mus	GACTGGGGACAGTTTTGAGAC
ROCK1-1 R QP-Mus	ATCCAAATCATAAACCAGGGCAT
ROCK2-1 F QP-Mus	GGTTTACAGATGAAAGCGGAAGA
ROCK2-1 R QP-Mus	GTGATGCCTTATGACGAACCAA
GAPDH-1 F QP-Mus	AGGTCGGTGTGAACGGATTTG
GAPDH-1 R QP-Mus	GGGGTCGTTGATGGCAACA
