# Supplementary material for: CRISPR-CasRx-mediated disruption of Aqp1/Adrb2/Rock1/Rock2 genes reduces intraocular pressure and retinal ganglion cell damage in mice
Source: Nat Commun. 2024 Jul 30;15:6395. doi: 10.1038/s41467-024-50050-4 (PMC11289368; doi:10.1038/s41467-024-50050-4)
Supplement: Supplementary file 3 — Reporting summary [file 41467_2024_50050_MOESM3_ESM.pdf]

nature portfolio

Corresponding author(s): Xingtao Zhou, Jinhai Huang

Last updated by author(s): Jun 5, 2024

Reporting Summary

Nature Portfolio wishes to improve the reproducibility of the work that we publish. This form provides structure for consistency and transparency in reporting. For further information on Nature Portfolio policies, see our Editorial Policies and the Editorial Policy Checklist.

Statistics

For all statistical analyses, confirm that the following items are present in the figure legend, table legend, main text, or Methods section.

n/a	Confirmed	
☐	☒	The exact sample size (*n*) for each experimental group/condition, given as a discrete number and unit of measurement
☐	☒	A statement on whether measurements were taken from distinct samples or whether the same sample was measured repeatedly
☐	☒	The statistical test(s) used AND whether they are one- or two-sided<br>*Only common tests should be described solely by name; describe more complex techniques in the Methods section.*
☒	☐	A description of all covariates tested
☐	☒	A description of any assumptions or corrections, such as tests of normality and adjustment for multiple comparisons
☐	☒	A full description of the statistical parameters including central tendency (e.g. means) or other basic estimates (e.g. regression coefficient) AND variation (e.g. standard deviation) or associated estimates of uncertainty (e.g. confidence intervals)
☐	☒	For null hypothesis testing, the test statistic (e.g. *F*, *t*, *r*) with confidence intervals, effect sizes, degrees of freedom and *P* value noted<br>*Give P values as exact values whenever suitable.*
☒	☐	For Bayesian analysis, information on the choice of priors and Markov chain Monte Carlo settings
☒	☐	For hierarchical and complex designs, identification of the appropriate level for tests and full reporting of outcomes
☒	☐	Estimates of effect sizes (e.g. Cohen's *d*, Pearson's *r*), indicating how they were calculated

Our web collection on statistics for biologists contains articles on many of the points above.

Software and code

Policy information about availability of computer code

Data collection	No software was used.
Data analysis	RGCs were counted using the software ImageJ (https://imagej.net/ij/index.html).The intensity of fluorescence signal was analyzed by ImageJ(https://imagej.net/ij/index.html).Statistical analysis and graphical representations were performed using GraphPad Prism (GraphPad PRISM, Version 8.0).Statistical analysis of IOP were performed using IBM SPSS Statistics 26 (Chicago, USA).<br>Single cell RNA sequence data was analyzed using R 4.2.0. The following R packages are used for analyzing scRNA-seq data: dplyr 1.1.1, Seurat 4.3.0.1, patchwork 1.1.2, SeuratData 0.2.2, ggplot2 3.4.2, DoubletFinder 2.0.3, harmony 0.1.1, clusterProfiler 4.6.2, AUCell 1.20.1, tidyr 1.3.0, reshape2 1.4.4, ComplexHeatmap 2.14.0, BuenColors 0.5.6, RColorBrewer 1.1-3, org.Mm.eg.db, 3.16.0, ggrepel 0.9.3, homologene 1.4.68.19.3.27, psych 2.3.3, qgraph 1.9.4, igraph 1.5.0.1, purrr 1.0.1, stringi 1.7.12, stringr 1.5.0, loomR 0.2.1.9000 and SeuratDisk 0.0.0.9020<br>The pySCENIC pipeline was utilized to perform SCENIC analysis.

For manuscripts utilizing custom algorithms or software that are central to the research but not yet described in published literature, software must be made available to editors and reviewers. We strongly encourage code deposition in a community repository (e.g. GitHub). See the Nature Portfolio guidelines for submitting code & software for further information.

Data

Policy information about availability of data

All manuscripts must include a data availability statement. This statement should provide the following information, where applicable:

- Accession codes, unique identifiers, or web links for publicly available datasets
- A description of any restrictions on data availability
- For clinical datasets or third party data, please ensure that the statement adheres to our policy

The single-cell RNA sequencing data generated in this study have been deposited in the NCBI GEO database. (https://www.ncbi.nlm.nih.gov/geo/query/acc.cgi?acc=GSE247776)

Research involving human participants, their data, or biological material

Policy information about studies with human participants or human data. See also policy information about sex, gender (identity/presentation), and sexual orientation and race, ethnicity and racism.

Reporting on sex and gender	*Use the terms sex (biological attribute) and gender (shaped by social and cultural circumstances) carefully in order to avoid confusing both terms. Indicate if findings apply to only one sex or gender; describe whether sex and gender were considered in study design; whether sex and/or gender was determined based on self-reporting or assigned and methods used. Provide in the source data disaggregated sex and gender data, where this information has been collected, and if consent has been obtained for sharing of individual-level data; provide overall numbers in this Reporting Summary. Please state if this information has not been collected. Report sex- and gender-based analyses where performed, justify reasons for lack of sex- and gender-based analysis.*
Reporting on race, ethnicity, or other socially relevant groupings	*Please specify the socially constructed or socially relevant categorization variable(s) used in your manuscript and explain why they were used. Please note that such variables should not be used as proxies for other socially constructed/relevant variables (for example, race or ethnicity should not be used as a proxy for socioeconomic status). Provide clear definitions of the relevant terms used, how they were provided (by the participants/respondents, the researchers, or third parties), and the method(s) used to classify people into the different categories (e.g. self-report, census or administrative data, social media data, etc.) Please provide details about how you controlled for confounding variables in your analyses.*
Population characteristics	*Describe the covariate-relevant population characteristics of the human research participants (e.g. age, genotypic information, past and current diagnosis and treatment categories). If you filled out the behavioural & social sciences study design questions and have nothing to add here, write "See above."*
Recruitment	*Describe how participants were recruited. Outline any potential self-selection bias or other biases that may be present and how these are likely to impact results.*
Ethics oversight	*Identify the organization(s) that approved the study protocol.*

Note that full information on the approval of the study protocol must also be provided in the manuscript.

Field-specific reporting

Please select the one below that is the best fit for your research. If you are not sure, read the appropriate sections before making your selection.

☒ Life sciences ☐ Behavioural & social sciences ☐ Ecological, evolutionary & environmental sciences

For a reference copy of the document with all sections, see nature.com/documents/nr-reporting-summary-flat.pdf

Life sciences study design

All studies must disclose on these points even when the disclosure is negative.

Sample size	We used a sample size of 2-34 samples. No sample size calculation was performed. We confirmed the sample size based on other studies in the references and statistical requirements.
Data exclusions	For RGCs counting, data were excluded if the sample quality is poor such as sample defect. No data were excluded from other analyses.
Replication	In order to verify the reproducibility of experimental results, all experiments except the single-cell RNA sequencing experiment were repeated for at least 3 times or at least 3 samples. all attempts at replication were successful.
Randomization	All samples were randomly grouped.
Blinding	In the intraocular pressure measurement experiment, investigators are blind to all groups allocation during data collection and analysis. In other experiments, the investigators were not blinded to allocation during experiments and outcome assessment.

Reporting for specific materials, systems and methods

We require information from authors about some types of materials, experimental systems and methods used in many studies. Here, indicate whether each material, system or method listed is relevant to your study. If you are not sure if a list item applies to your research, read the appropriate section before selecting a response.

Materials & experimental systems

n/a	Involved in the study
☐	☒ Antibodies
☐	☒ Eukaryotic cell lines
☒	☐ Palaeontology and archaeology
☐	☒ Animals and other organisms
☒	☐ Clinical data
☒	☐ Dual use research of concern
☒	☐ Plants

Methods

n/a	Involved in the study
☒	☐ ChIP-seq
☐	☒ Flow cytometry
☒	☐ MRI-based neuroimaging

Antibodies

Antibodies used

Primary antibodies used:
rabbit anti-beta 2 adrenergic receptor (1:100; Cat#ab182136; Abcam, UK),
rabbit anti-aquaporin 1(1:500; Cat#ab300463; Abcam, UK),
ROCK1 (E2G4N) Rabbit mAb (1:50; Cat#28999S; Cell Signaling Technology, USA),
ROCK2 (E5T5P) Rabbit mAb (1:50; Cat#47012S; Cell Signaling Technology, USA),
RCVRN Rabbit pAb (1:200; Cat#A6404;ABclonal, China) ,
anti-Arrestin-C (1:200; Cat#ab15282; Sigma-Aldrich, USA),
RBPMS rabbit polyclonal antibody (1:100; Cat#15187-1-AP; Proteintech, USA)
mouse anti-Flag (1:4000; Cat#F3165; Sigma-Aldrich, USA).

Secondary antibodies used:
Alexa Fluor Cy3 AffiniPure Donkey Anti-Rabbit IgG (1:400; Cat#160185; Jackson ImmunoResearch Labs, USA)
Alexa Fluor Cy5 AffiniPure Donkey Anti-Mouse IgG (1:400; Cat#145170; Jackson ImmunoResearch Labs, USA).
Alexa Fluor 488 AffiniPure Donkey Anti-Rabbit IgG (1:200; Cat# 156556; Jackson ImmunoResearch Labs, USA)

Validation

Antibodies were from commercial and validated for each application by the manufactures. Multiple dilutions were tested to determine the most appropriate dilution.
rabbit anti-beta 2 adrenergic receptor (Cat#ab182136; Abcam, UK): https://www.abcam.com/products/primary-antibodies/beta-2-adrenergic-receptor-antibody-epr707n-ab182136.html
rabbit anti-aquaporin 1(Cat#ab300463; Abcam, UK): https://www.abcam.com/products/primary-antibodies/aquaporin-1-antibody-epr26554-46-ab300463.html
ROCK1 (E2G4N) Rabbit mAb (Cat#28999S; Cell Signaling Technology, USA): https://www.cellsignal.com/products/primary-antibodies/rock1-e2g4n-rabbit-mab/28999
ROCK2 (E5T5P) Rabbit mAb (Cat#47012S; Cell Signaling Technology, USA): https://www.cellsignal.com/products/primary-antibodies/rock2-e5t5p-rabbit-mab/47012
RCVRN Rabbit pAb (Cat#A6404;ABclonal, China): https://abclonal.com.cn/catalog/A6404
anti-Arrestin-C (Cat#ab15282; Sigma-Aldrich, USA): https://www.sigmaaldrich.com/HK/zh/product/mm/ab15282
RBPMS rabbit polyclonal antibody (Cat#15187-1-AP; Proteintech, USA) :https://www.ptgcn.com/products/RBPMS-Antibody-15187-1-AP.htm
mouse anti-Flag (Cat#F3165; Sigma-Aldrich, USA):https://www.sigmaaldrich.com/HK/zh/product/sigma/f3165

Eukaryotic cell lines

Policy information about cell lines and Sex and Gender in Research

Cell line source(s)

N2a cells are mouse neuroblasts with neuronal and amoeboid stem cell morphology isolated from mouse brain tissue.

Authentication

The N2a cell line was purchased from the cell bank of the Shanghai Institute of Biochemistry and Cell Biology, Chinese Academy of Sciences.

Mycoplasma contamination

We confirmed that all cell lines tested negative for mycoplasma contamination.

Commonly misidentified lines
(See ICLAC register)

The study did not involved commonly misidentified lines.

Animals and other research organisms

Policy information about studies involving animals; ARRIVE guidelines recommended for reporting animal research, and Sex and Gender in Research

Laboratory animals	Eight-week-old C57BL/6J female mice used in this study were purchased from Vital River Laboratories Beijing, China.
Wild animals	The study did not involved wild animals.
Reporting on sex	According to several literature sources, sex has been reported to exert an influence on intraocular pressure in glaucoma. To mitigate this potential impact, we conducted our experiments exclusively using mice of a only one sex.
Field-collected samples	The study did not involved samples collected from field.
Ethics oversight	All animal experiments were approved by the Animal Care and Use Committee of Fudan University, Shanghai, China.

Note that full information on the approval of the study protocol must also be provided in the manuscript.

Plants

Seed stocks	*Report on the source of all seed stocks or other plant material used. If applicable, state the seed stock centre and catalogue number. If plant specimens were collected from the field, describe the collection location, date and sampling procedures.*
Novel plant genotypes	*Describe the methods by which all novel plant genotypes were produced. This includes those generated by transgenic approaches, gene editing, chemical/radiation-based mutagenesis and hybridization. For transgenic lines, describe the transformation method, the number of independent lines analyzed and the generation upon which experiments were performed. For gene-edited lines, describe the editor used, the endogenous sequence targeted for editing, the targeting guide RNA sequence (if applicable) and how the editor was applied.*
Authentication	*Describe any authentication procedures for each seed stock used or novel genotype generated. Describe any experiments used to assess the effect of a mutation and, where applicable, how potential secondary effects (e.g. second site T-DNA insertions, mosiacism, off-target gene editing) were examined.*

Flow Cytometry

Plots

Confirm that:

☒ The axis labels state the marker and fluorochrome used (e.g. CD4-FITC).

☒ The axis scales are clearly visible. Include numbers along axes only for bottom left plot of group (a 'group' is an analysis of identical markers).

☒ All plots are contour plots with outliers or pseudocolor plots.

☒ A numerical value for number of cells or percentage (with statistics) is provided.

Methodology

Sample preparation	To isolate cells, the transfected or non-transfected were dissociated enzymatically in an incubation solution of 100 ul Trypsin-EDTA (0.05%) at 37°C for 3 min. The digestion was stopped by adding 1 ml of DMEM medium with 10% Fetal Bovine Serum (FBS). The cell suspension was centrifuged for 3 min (1000 pm), and the pellet was resuspended in DMEM medium with 10% FBS. Finally. the cell suspension was filtered through a 40-um cell strainer. and mCherry+/EGFP+ cells were isolated.
Instrument	BD FACSAriaTM Fusion cytometry
Software	FlowJo V10.5.3
Cell population abundance	Samples were found to be >95% pure when assessed with a second round of flow cytometry analysis.
Gating strategy	1) in FSC-A/SSC-A gate for living cells, 2) using the non-transfected cells to define the gate for EGFP+ and/or mCherry+ cells, 3) apply this gate to all samples.

☒ Tick this box to confirm that a figure exemplifying the gating strategy is provided in the Supplementary Information.
