# Supplementary material for: CRISPR-CasRx-mediated disruption of Aqp1/Adrb2/Rock1/Rock2 genes reduces intraocular pressure and retinal ganglion cell damage in mice
Source: Nat Commun. 2024 Jul 30;15:6395. doi: 10.1038/s41467-024-50050-4 (PMC11289368; doi:10.1038/s41467-024-50050-4)
Supplement: Supplementary file 4 — Table S3 [file 41467_2024_50050_MOESM4_ESM.pdf]

Table S3：List of primers used for qPCR

AQP1-1 F QP-Mus	AGGCTTCAATTACCCACTGGA
AQP1-1 R QP-Mus	CTTTGGGCCAGAGTAGCGAT
ADRB2-1 F QP-Mus	ATGTCGGTTATCGTCCTGGC
ADRB2-1 R QP-Mus	GGTTTGTAGTCGCTCGAACTTG
ROCK1-1 F QP-Mus	GACTGGGGACAGTTTTGAGAC
ROCK1-1 R QP-Mus	ATCCAAATCATAAACCAGGGCAT
ROCK2-1 F QP-Mus	GGTTTACAGATGAAAGCGGAAGA
ROCK2-1 R QP-Mus	GTGATGCCTTATGACGAACCAA
GAPDH-1 F QP-Mus	AGGTCGGTGTGAACGGATTTG
GAPDH-1 R QP-Mus	GGGGTCGTTGATGGCAACA
